# Bamboo Fiber Based Cellulose Nanocrystals/Poly(Lactic Acid)/Poly(Butylene Succinate) Nanocomposites: Morphological, Mechanical and Thermal Properties

**DOI:** 10.3390/polym13071076

**Published:** 2021-03-29

**Authors:** Masrat Rasheed, Mohammad Jawaid, Bisma Parveez

**Affiliations:** 1 Laboratory of Biocomposite Technology, Institute of Tropical Forestry and Forest Products (INTROP), Universiti Putra Malaysia, Serdang 43400, Selangor, Malaysia; masuqayyuum@gmail.com; 2 Kulliyan of Engineering (KOE), Islamic International University Malaysia, Gombak 53100, Kuala Lumpur, Malaysia; mirbisma5555@gmail.com

**Keywords:** poly(lactic acid), poly(butylene succinate), cellulose nanocrystals (CNC), nanocomposites: morphological properties

## Abstract

The purpose of this work was to investigate the effect of cellulose nanocrystals (CNC) from bamboo fiber on the properties of poly (lactic acid) (PLA)/poly (butylene succinate) (PBS) composites fabricated by melt mixing at 175 °C and then hot pressing at 180 °C. PBS and CNC (0.5, 0.75, 1, 1.5 wt.%) were added to improvise the properties of PLA. The morphological, physiochemical and crystallinity properties of nanocomposites were analysed by field emission scanning electron microscope (FESEM), Fourier-transform infrared spectroscopy (FTIR) and X-ray diffractometry (XRD), respectively. The thermal and tensile properties were analysed by thermogravimetic analysis (TGA), Differential scanning calorimetry (DSC) and Universal testing machine (UTM). PLA-PBS blend shows homogeneous morphology while the composite shows rod-like CNC particles, which are embedded in the polymer matrix. The uniform distribution of CNC particles in the nanocomposites improves their thermal stability, tensile strength and tensile modulus up to 1 wt.%; however, their elongation at break decreases. Thus, CNC addition in PLA-PBS matrix improves structural and thermal properties of the composite. The composite, thus developed, using CNC (a natural fiber) and PLA-PBS (biodegradable polymers) could be of immense importance as they could allow complete degradation in soil, making it a potential alternative material to existing packaging materials in the market that could be environment friendly.

## 1. Introduction

The increasing usage of petroleum-based plastics in the present times is leading to increased accumulation of plastic waste in the environment as their natural degradation time is longer. To prevent this, there is a need to develop environment friendly bioplastics derived from biomass. PLA, a bioplastic material that originates from the renewable resources, is mostly preferred and extensively studied as environmental and sustainable material [1]. It has high strength, high modulus and good clarity. However, PLA is brittle, with low toughness, slow degradation, slow rate of crystallisation and elongation at break less than 10% that restrict its usage [2]. These properties can be enhanced by adding fillers or additives [3]. Moreover, its mechanical properties can be improvised by blending with PBS. On the other hand, PBS is flexible and tough; when blended with PLA, it overcomes the limitations of PLA and results in the development of plastic materials of desirable mechanical properties [4,5,6]. Additionally, the ability of PLA-PBS blend for packaging application has been reported earlier by the researchers [7] and higher miscibility has also been achieved [8]. Further, the addition of natural fibers (bamboo fiber) in the blend has proved to improve their properties; however, there was an improvement in tensile strength [9,10]. Furthermore, cellulose nanocrystals (CNCs) incorporation in the blend can improve their properties more significantly, as in case of [11]. CNCs are also derived from renewable resources and have low density, exceptional morphology, high aspect ratio, higher mechanical strength, good biodegradability and larger surface area [12]. CNCs are obtained from cellulose, which is abundantly available in the nature.CNCs can be produced via several techniques mostly by oxidation using ammonium persulfate or by hydrolysis of microcrystalline cellulose (MCC) using sulphuric acid [13,14,15].

Adding cellulose to the biocomposites increases their mechanical strength [16]. At nanoscale, cellulose can considerably modify the chemical or physical properties of nanocomposites [17]. CNC has been added to PLA by many researchers, showing that mechanical properties of the developed PLA/CNC nanocomposites, fabricated via solvent exchange method, get enhanced [18]. Some other methods used to prepare the composite include melt blending method [19]. Further, Pirani [20] investigated the mechanical properties of electro spun PLA-CNC composite developed using aqueous mixing and freeze-drying techniques. CNCs have also been reinforced in PBS by various researchers [21,22]. Thus, it is interesting to determine the influence of PBS and CNC together, on the properties of the PLA. Luzi et al. [23] reported an improved barrier property as a result of collective nucleation effect of CNCs extracted from hemp fiber and PBS content about 20% on PLA matrix, thereby improving its crystallinity. There is not much research work dedicated to the study of CNC-reinforced PLA composites as it is hard to get the uniform distribution of nanoparticles in the polymer matrix [24]. To explore this area further, the CNCs extracted from bamboo fiber will be incorporated in PLA-PBS polymer blend to enhance their properties further and achieve uniform distribution of CNCs in the PLA-PBS blends.

In this research work, the main aim was to investigate the impact of PBS and cellulose nanocrystals (CNC) on the properties of PLA based nanocomposite. The CNCs were synthesised by acid hydrolysis technique. Then, the extracted CNCs were used as reinforcements to fabricate PLA-PBS-CNC nanocomposites by using hot pressing technique. The composites were further characterised to analyse the effect of the PBS and CNCs on properties of the nanocomposites. SEM, XRD, FTIR and tensile testing were carried out for investigating the morphology, crystallinity, physiochemical and mechanical properties of the fabricated composites. This work contributes towards the attainment of better dispersion of CNCs in the PLA-PBS blend thereby enhancing their properties and thus advancing our understanding in promoting the development of biocomposites as replacement for the conventional plastics and material for packing applications.

## 2. Materials and Methods

### 2.1. Materials

PLA pellets (7001 Ingeo^TM^ of specific gravity 1.24) were obtained from Nature Works LCC, MN, USA. It has a melting point of 154 °C and is a hydrophobic polymer. PBS pellets (1.26 g/cm^3^) were procured from PTT public company limited in Bangkok, Thailand. The melting point of PBS is 95 °C. CNCs were extracted from bamboo fiber via acid hydrolysis technique. The average length of isolated CNCs was 200–500nm and an average diameter of 10 nm [25].

### 2.2. Methods

#### 2.2.1. Extraction of CNC from Bamboo Fiber

Bamboo chips were dried in oven and pretreated before isolation by NaOH (17 wt.%) aqueous solution, followed by 40% of sodium hypochlorite and lastly with NaOH to eliminate the residual lignin, fatty acids and other impurities, and also to remove the hemicellulose and lignin from bamboo pulp, respectively. In addition, there was a swelling of amorphous cellulose during the pre-treatment in order to facilitate the penetration of sulfuric acid (64%,100 mL) into the bamboo pulp during the process of acid hydrolysis executed at 45 °C for 45 min, and was continuously stirred at the constant frequency of 20 kHz in an ultrasonic bath. Further, when there was a change in suspension colour into dark yellow, then the hydrolysis was discontinued upon addition of deionised water (DI) and left for several hours until layered suspensions were obtained and were decanted off until no more layer appeared. Then, the suspension was washed with DI water and subjected to centrifuge cycles at the speed of 6000 rpm for the duration of 15 min. The suspension was washed out, and again DI water was added, and this process was discontinued until it turned turbid. Finally, it was washed many times until pH of water did not vary and the extracted CNC were then dried using oven at 55 °C for the duration of 24 h and were obtained in the form of powder [25].

#### 2.2.2. Preparation of CNC Reinforced PLA/PBS Composites

PLA, PBS and CNC were dried at 60 °C for 24 h using an oven. Six compositions as mentioned in Table 1 were prepared by hot pressing technique. Initially the constituents of compositions were mixed by melt mixer at the temperature of 175 °C at 60 rpm for 10 min. The mixture was then crushed in a crusher to get it in the form of pellets. Compressed nano-composite films with a thickness of 0.09mm were obtained by compressing these pellets in a hot press at the temperature of 180 °C at the constant pressing pressure of 150 MPa for reheating time of 5 min and then pressing time of 4 min. The compressed sheets were then dried in oven at 50 °C for 24 h. The fabricated samples were stored in desiccator prior to testing.

### 2.3. Characterisation and Testing

#### 2.3.1. Field Electron Scanning Electron Microscopy (FESEM)

The morphologies of nanocomposite films were examined via JEOL JSM-7000F FESEM (Tokyo, Japan) at 10–20 kV (accelerating voltage). To prevent electrostatic charging of films, these were coated before inspection.

#### 2.3.2. Thermal Properties

Thermal stability of the nanocomposite films was determined by using Perkin-Elmer TGA7 (Wellesley, MA, USA). TGA analysis was executed on samples around 10 mg at 10 °C/min (heating rate) from 0 to 600 °C in the presence of nitrogen atmosphere. Then, the variation in weight ratio with respect to temperature of the samples was recorded. DSC analysis was carried via Perkin-Elmer DSC7 (Wellesley, MA, USA) using samples of around 3–5 mg in weight, which were placed in the sample pan followed by the performance of temperature scan at 10 °C/min (heating rate) in temperature range of 0 to 200 °C. Here, an aluminum pan was treated as a reference. From the scan results, the glass transition temperature (T_g_), crystallization temperature (T_c_) and melting temperature (T_m_) for nanocomposite films were revealed.

#### 2.3.3. XRD

XRD was performed to find a crystalline structure of nanocomposite films by SHIMADZU XRD-6000 (Tokyo Japan) employing Cu K-alpha radiation that were Ni-filtered and the incident angle ranging from of 20 °C to 70 °C.

#### 2.3.4. FTIR

FTIR spectra were obtained using FTIR (Perkin Elmer 1600 Infrared spectrometer, Wellesley, MA, USA) along with a universal attenuated total reflection technique. The spectra of the composite films were measured in the frequency range of 500–4000 cm^−1^.

#### 2.3.5. Tensile Properties

Tensile properties were acquired as per ASTM Standard Method (D638-14 (2014)) via (Lloyds LRX) UTM (Largo, FL, USA). The nanocomposite films of dimensions (10 mm × 100 mm) were used, and tensile testing was executed at 10 mm/min crosshead speed and 50 mm as initial grip separation. Ten samples of each composition were dried at 50 °C for 24 h before testing. Then, the tensile strength, elongation at break and tensile modulus were acquired from stress-strain curve. Tests were repeated for four samples of each composition to get the accuracy in results.

#### 2.3.6. SEM

The fractured surface of tensile tested films was studied via Hitachi Model S-3400N SEM (Tokyo, Japan). The fractured surface was coated with gold by sputter coating process before examination to prevent electrostatic charging.

## 3. Results and Discussion

### 3.1. Morphology

**Figure 1:**
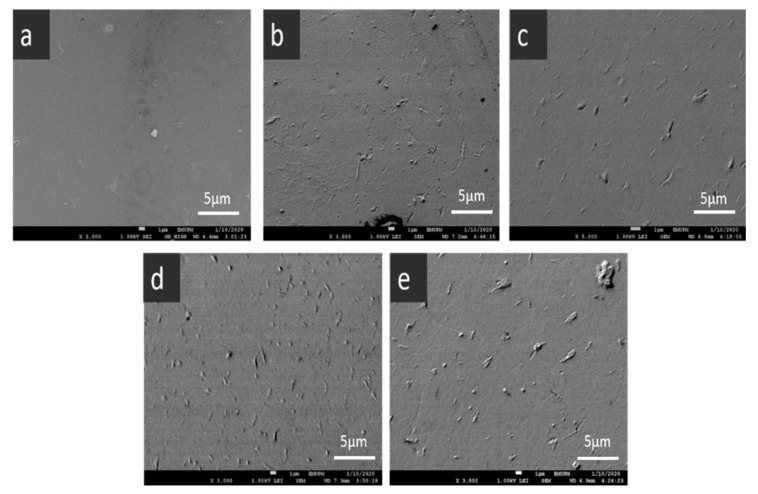
FESEM images of PLA/PBS composites, (**a**) N1, (**b**) N2, (**c**) N3, (**d**) N4and (**e**) N5.

FESEM was utilised to analyse the morphology of developed PLA-PBS and PLA-PBS-CNC nanocomposite films, and the distribution of CNCs in the PLA-PBS matrix. Figure 1a reveals the smooth surface of PLA-PBS blend also displaying their homogeneous morphology, and Figure 1b–e shows the composites containing rod-like CNC particles that are embedded in the polymer surface. As from our previous study, the dimensions of extracted CNCs from TEM and AFM were typically ranging from 200–500 nm (length) and 10 nm(diameter) [25]. In addition, composites (N2, N3, N4 and N5) reveal quite uniform dispersion of CNC in the nanocomposites up to 1 wt.%, as evident from Figure 1b–e. Therefore, the CNCs were approximately homogeneously distributed inside the matrix polymer as the presence of micro agglomerates of nanocellulose were not revealed from the FESEM images [26]. On further addition of CNC, micro agglomerates were found as evident from Figure 1e. Thus, better dispersion of CNCs were obtained in the resultant composites.

### 3.2. Thermal Properties

**Figure 2:**
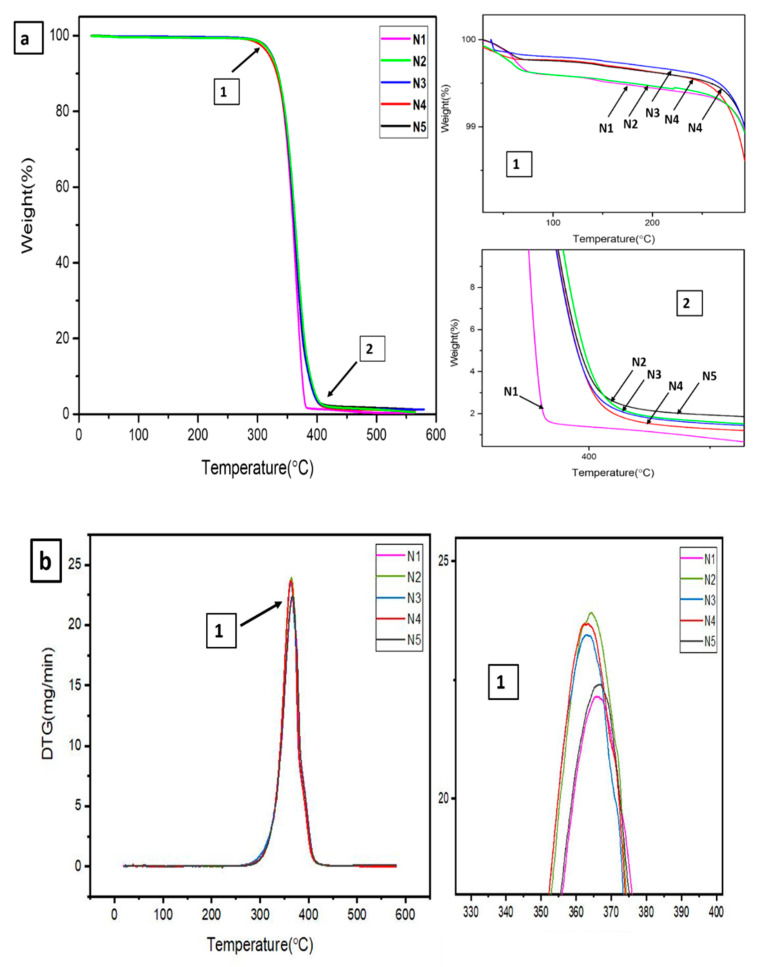
(**a**) TGA and (**b**) DTG of PLA/PBS, PLA/PBS/CNC composites.

To determine the thermal stability of the nanocomposite films, the TGA and DTG curves for PLA-PBS and PLA-PBS-CNC (0.5, 0.75, 1 and 1.5 wt.%) composite films were acquired as demonstrated in Figure 2 and their results were given in Table 2. The initial thermal decomposition of PLA-PBS was observed at 331.7 °C. However, for CNC-reinforced composites, the initial thermal degradation temperature upon addition of CNC decreased (331.2 °C) first, then it increased (334 and 332 °C) for 0.75 and 1 wt.% of CNC, respectively [27].On further addition, it decreased to a value of 330 °C, as evident from Figure 2a. The maximum degradation temperature is the highest degradation peak demonstrated by DTG thermogram [28]. The maximum degradation temperature (Tmax) was obtained from DTG; it first increased by adding CNC to the PLA-PBS matrix. The Tmax of the blend is higher, that is, 366.08, then decreased insignificantly, and on further addition it increased to 367.52 and the weight loss at maximum temperature increased insignificantly due to presence of CNC, as shown in Figure 2b.

However, final degradation temperature was higher in case of PLA-PBS composite (396.8 °C), upon addition of CNC, it was reduced (387.3 °C) initially then it increased (390 and 391 °C for 0.75 and 1 wt.% of CNC, respectively). On further addition it decreased (385.2 °C). Thus, Tf and T50% increased on incorporation of CNC in the PLA-PBS matrix, indicating that CNC improves the thermal stability of PLA and retards the thermal degradation rate [29]. Comparable results were observed by [30] for polyvinyl alcohol-based nanocellulose composites and by [31] on the PLA/wood-based CNC nanocomposites. Additionally, the higher value of thermal degradation temperature can be credited to the interaction of CNC with PLA-PBS matrix, impeding chain movement and preventing melting of chain during the process of degradation [32,33,34]. In case of weight residue for PLA-PBS composite, the same trend was observed, where it was found to be least (0.562%); however, upon addition of CNC, it increased (1.172%), then on further addition upto 1wt.% it decreased to 0.3676% and 1.068% for 0.75 and 1 wt.% of CNC, respectively.

**Figure 3:**
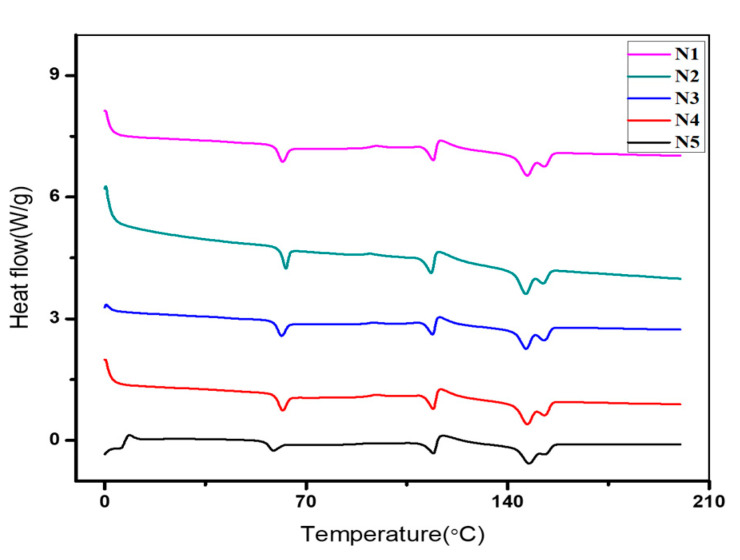
DSC of PLA-PBS, PLA-PBS-CNC composite films.

DSC analysis was performed to explore how CNC incorporation affects the thermal properties of nanocomposites. The DSC curves of the PLA-PBS and nanocomposites with CNCs are demonstrated in Figure 3. The crystallinity and melting parameters as acquired from the DSC curves are mentioned in Table 3. From the curves of PLA-PBS and their nanocomposites, glass transition peak and melting peak were observed at around 58.49 and 114.1 corresponding to the PLA and PBS, respectively [26,35]. Further, the double melting peaks were witnessed at 147.2 and 153.59 °C. The double melting peaks implies the thickness of two lamellas; the thinner and the thicker one melts at low and higher temperature, respectively [36,37]. Many researchers [38,39,40,41] described this Tm as a presence of α and α’. In addition, the two temperatures indicate the melting temperatures of unstable and stable crystals that melt at low and high temperatures, respectively. Thus, the melting rate was found to be greater than the crystallisation rate, and result in an endothermic peak [42].

Here, PBS restricts crystallization with similar findings as the crystallization behaviour observed in the case of other polymer blends [43,44]. PBS as well as the CNC content in the blend increased the melting temperature of PLA in the blend. Additionally, the sharper and narrower peaks were obtained upon addition of CNC up to 1wt.% (N2, N3 and N4), and on further addition broadened peaks were obtained as in case of composite with 1.5wt.% CNC(N5). It could be assumed that the CNCs in the composites N2, N3 and N4 are more homogeneously distributed in the blend. The nucleation effect of CNC on PLA was reduced due to existence of PBS in the blend as PBS inhibits the crystallisation of PLA in the blends, indicating that the interaction of PBS with CNC is stronger in comparison to PLA with CNCs [45]. Hence, PBS covers most of the surfaces of CNCs and restricts the non-uniform nucleation effect of PLA by CNC. Similar behavior was noticed in the PVDF-PBS-CNT and PLA-PBS-CNC systems fabricated via solution casting techniques [46,47]. Thus, thermal analysis of nano composites revealed enhancement upon addition of CNC, and similar improvement was achieved in the previous work where CNCs extracted from hemp fibers were added in PLA-PBS composites [23].

### 3.3. X-ray Diffractometer (XRD)

**Figure 4:**
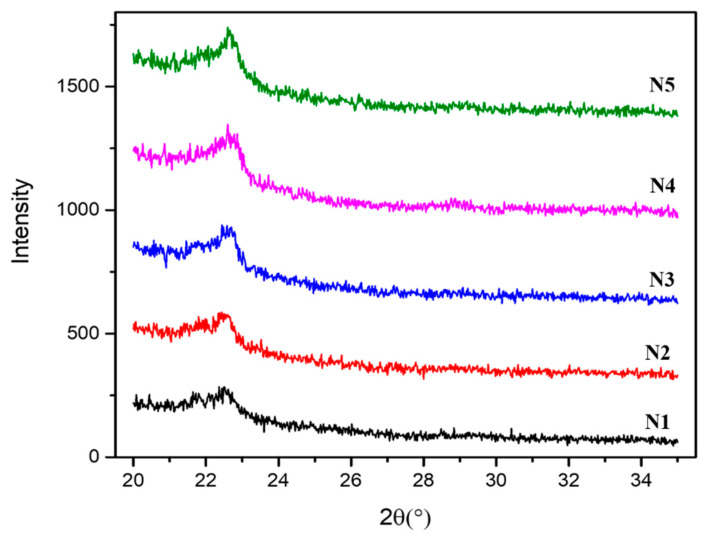
XRD of PLA-PBS, PLA-PBS-CNC nanocomposite films.

The crystalline structure of the nanocomposite films and the influence of CNC on the crystal structure of the composite films N1–N5 were examined by the XRD analysis as shown in Figure 4. Sharper peaks were obtained on inclusion of CNC, thereby influencing the crystallinity of the composites, as evident from Figure 4. Comparable findings were acquired when CNC was added to PLA-based nanocomposites [20]. The CNC used as extracted from bamboo fiber revealed sharper peak at 22.8°, indicating the highest crystallinity degree of 86.96% as reported in our previous work [25]. The PLA component in the composites revealed broad and obscure diffraction patterns [48,49], indicating the existence of PLA mostly in amorphous state with small number of crystallites as observed in our previous work for pure PLA composite developed using the same parameters and techniques [50]. This indicates lower rate of crystallisation of PLA, which may be due to the fast rate of cooling during processing, preventing crystallisation of PLA [51,52]. PBS exhibits the main distinctive diffraction peaks at 2θ; 19.6, 21.8 and 22.7 corresponding to the (020), (021) and (110) planes of PBS, respectively [53,54].

Addition of the PBS in the PLA causes a rise in the peak intensities. Moreover, there was insignificant co-crystallisation at the interfaces of PLA and PBS [55] as also evident from DSC curve in Figure 3. The absence of PLA diffraction peak in all the five composites (N1–N5) indicates that the PLA component has undergone no crystallisation, while the diffraction peak of PBS at 22.5° was noticed in all composites. Similar diffraction patterns were obtained for PLA-PBS-CNC nanocomposite films, indicating that the retention of the crystal structure of PBS was on incorporation of CNC, irrespective of the CNC contents [42]. In addition, the PBS peak intensity increased upon increasing CNC content. However, this can be credited to the integration of the diffraction peak of PBS with the CNC diffraction peak at 2ϴ = 23° [56]. Due to this, it was hard to differentiate the change of crystalline properties in the composites. Thus, the addition of PBS and CNC does not undergo crystallisation of PLA, thereby their crystal structure remains the same.

### 3.4. Physiochemical Analysis

**Figure 5:**
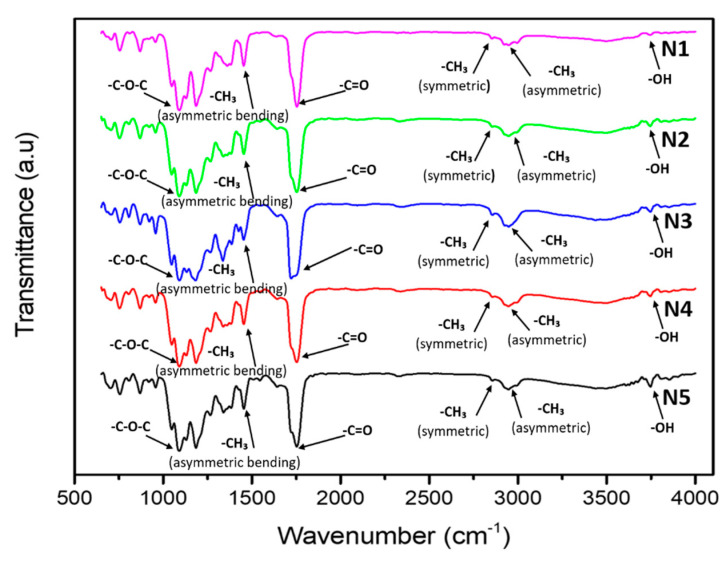
FTIR of PLA-PBS, PLA-PBS-CNC nanocomposite films.

The FTIR analysis was obtained to examine the chemical structure of the PLA-PBS(N1), and PLA-PBS-CNC (N2–N5) composites, with varying CNC content as presented in Figure 5. The FTIR spectrum of PLA-PBS-CNC (Figure 5) revealed the characteristic peaks of OH stretching vibration at 3502 cm^−1^, 2994 and 2955 cm^−1^ [57]. The asymmetric and symmetric stretching vibrations of CH3, respectively, were observed, with peak at 1753 cm^−1^ that indicated the stretching vibration of C=O. The asymmetric bending vibration of CH3 was displayed by peak at 1455 cm^−1^ and peaks at 1186, 1086 and 1094 cm^−1^, referring to stretching vibrations of C–O–C [58] of PLA and PBS. Bending frequencies for –CH3 symmetric and –CH3 asymmetric were spotted at 1361 and 1452 cm^−1^, respectively [58]. The peaks at 754 and 868 cm^−1^ represent the crystalline and amorphous phases of PLA, respectively, which was also observed in the FTIR analysis of PLA in our previous work [50].

In the case of FTIR of CNC sample reported in our previous work, a strong band was seen at 3332 cm^−1^, 2899 cm^−1^, and 1636 cm^−1^, indicating the O–H stretching vibration and thereby revealing their hydrophilic nature, as well as the symmetric C–H vibrations, an intense adsorption originating from the absorbed water. The sharper peaks of CNC at 1732 cm^−1^ and 1048 cm^−1^ indicated the reduction of hemicellulose and lignin and C-O-C pyranose ring vibration, demonstrating higher quantity of cellulose in CNC [25].Similar characteristics peaks were reported in the literature [59,60]. Figure 5 demonstrates the broadening of the distinctive carbonyl peak at 1753 cm^−1^, implying the partial dispersion of PLA and PBS [61]. In addition, this behaviour may be due to C=O or C–O and the OH interactions at the chain endings of PLA and PBS. Furthermore, PLA-PBS and PLA-PBS-CNC nanocomposite present identical absorption peaks as that of PLA and PBS. This indicates that there exist definite interactions between the polymers and the CNCs and no chemical interactions or new bonds were formed within the nanocomposites [62].

### 3.5. Tensile Properties

**Figure 6:**
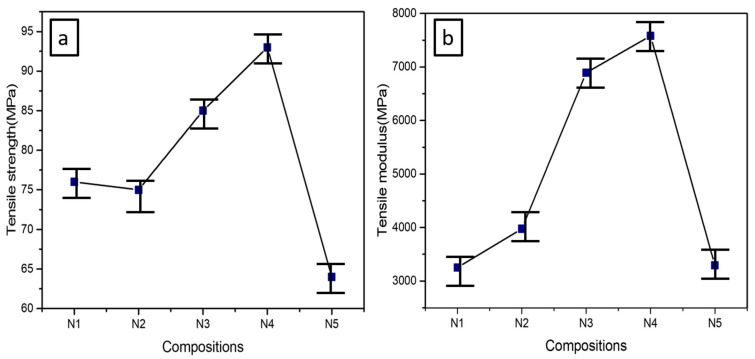
(**a**) Tensile strength (MPa) and (**b**) tensile modulus (MPa) of PLA-PBS-CNC nanocomposites at varying percentages of CNC.

**Figure 7:**
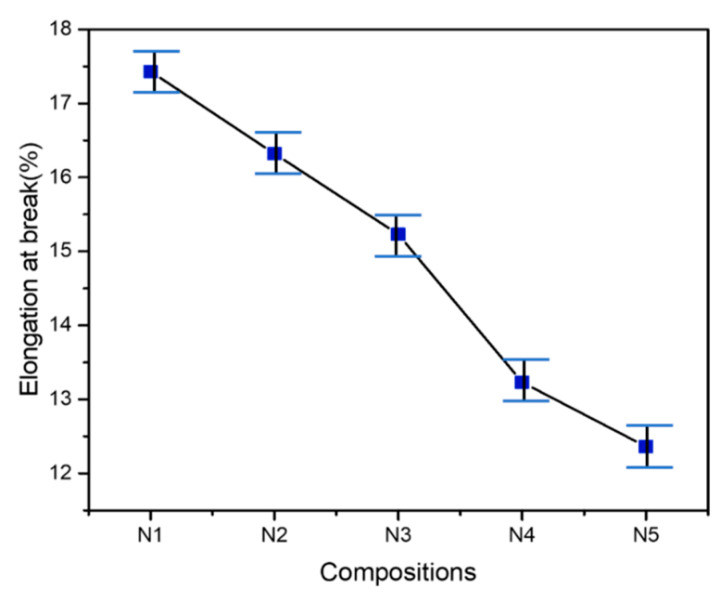
Elongation at break (%) of PLA-PBS-CNC nanocomposites at varying percentages of CNC.

The tensile testing was executed to evaluate the tensile strength (MPa), tensile modulus (MPa) and elongation at break(%). The tensile properties of PLA-PBS (N1) and PLA-PBS-CNC (N2–N5) composite films were tested at room temperature and tests were repeated four times as demonstrated by Table 4. The average, standard deviation and error values of the results were obtained on repetition to achieve accuracy. In addition, the average values of tensile strength (MPa), tensile modulus (MPa) and elongation at break (%) are plotted in Figure 6 and Figure 7.

The PLA-PBS has typically high tensile strength, tensile modulus and elongation at break. Similar results were reported in some previous works [23,58]. CNCs’ effect on the mechanical properties of PLA-PBS composite blend is presented in Figure 7. The purpose of CNC addition is to enhance mechanical properties of these composite films. Tensile strength of PLA-PBS composite enhances with the incorporation of CNCs, and it increases with increase in CNC content up to 1wt.% (N4); on further addition, tensile strength decreases, as evident from Figure 6a.

The enhancement of the tensile properties of the PLA-PBS-CNC composite films can be credited to the uniform distribution and placement of the CNCs in the matrix, as evident from Figure 1, and the strong interface interaction between them. However, the decreased values of tensile strength on incorporation of CNC above 1wt.% could be ascribed to intrinsic van der Waals interaction of CNC, which results in unavoidable agglomeration of the CNC, thereby inhibiting effective transfer of load to the matrix polymer [11]. Further, the introduction of higher content of nanoparticles may lead to the aggregation of CNCs and possible separation of microphase in the composites. In addition, the increased agglomeration of CNCs can further cause the reduction of mechanical properties of nanocomposites, which can be correlated with SEM images (Figure 7). Comparable results were also stated by other researchers [31,63].

In the meantime, tensile modulus also increases upon addition of CNC, as shown in Figure 6b, indicating the higher stiffness of the composites with CNC loading [29]. Further, upon increasing CNC content, it decreases due to agglomeration of CNCs that fail to offer strong interactions between the reinforcement and the matrix. Figure 7 shows the elongation at break of PLA-PBS-CNC nanocomposites. However, a slight decrease in elongation at break of PLA-PBS blend upon incorporation of CNC was observed, thus indicating the lower strain rates experienced by the composites upon addition of CNC. The reason for this can be associated with the CNCs aspect ratio being large and also the interaction at the interfaces between CNC and the matrix, constraining the motion of the polymer chains [52]. Thus, the incorporation of CNC improved the tensile strength and tensile modulus up to a certain limit; however, elongation at break was reduced upon addition of CNC in PLA-PBS composites.

### 3.6. SEM Analysis of Fractured Surfaces

**Figure 8:**
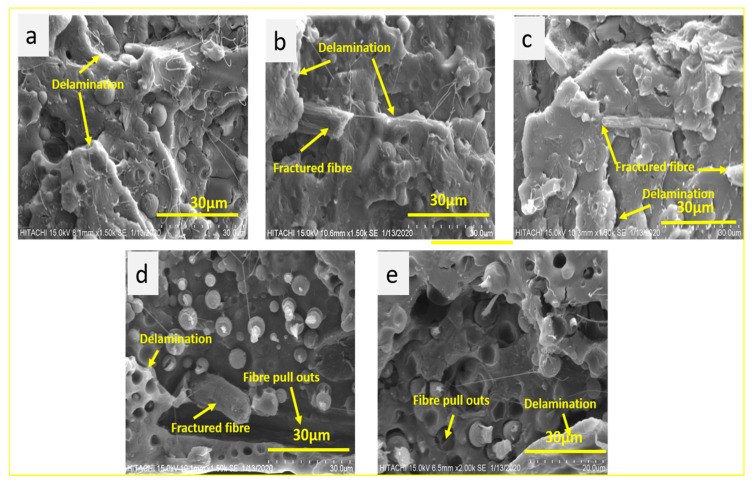
SEM morphology of the tensile tested (fractured) surfaces composites, (**a**) N1, (**b**) N2, (**c**) N3, (**d**) N4, (**e**) N5.

The relationship of mechanical properties with the comprehensive structures of the composites was studied using SEM micrography of the tensile tested PLA-PBS-CNC nanocomposite films with varying percentages of CNCs, as evident from Figure 8. The fractured surfaces are rougher with delaminations, broken fibers and some fiber pull-outs [64]. The broken fiber ends are mostly observed from the fractured surfaces, indicating a brittle failure mode [65].

PLA-PBS blend revealed clear interfaces between PLA and PBS in all the blends. This reveals the immiscibility of the blend [66,67]. When lower content of CNCs less than 1wt.% was introduced, the nanofibers get distributed uniformly in PLA-PBS matrix as demonstrated by Figure 8b–e. Subsequently, PLA-PBS-CNC nanocomposite films displayed rougher surfaces and the blends with relatively low CNC contents exhibited more interfacial bonding as a result. CNC fiber does not debond easily; rather, it undergoes brittle fracture during the fracture process, indicating that the matrix is adhered to the fibers, which reveals a good bonding at the interface of the fiber and matrix [68]. This leads to brittle failure in the fibers as interfacial bonding influences the interlaminar tensile strength, the interlaminar shear strength and the intralaminar strength [69] as evident from Figure 8b–d. However, blending 1.5 wt.% of CNCs (N5) with the PLA-PBS leads to formation of some fiber pull-outs as observed from composite fractured surfaces in Figure 8e. This fiber pull-out implies that the fiber-matrix interfacial bond strength surpassed before the tensile failure strength of the composite was acquired at this loading rate. The fiber pull-out is dependent on the bonding strength as well as on the load transfer mechanism from matrix to fiber [65]. The lower bonding strength in composite N5 with increased CNC content thereby reduced the mechanical strength. All fractured surfaces showed a matrix delamination and cracking, displaying the direction of crack propagation through the matrix as evident from Figure 8 [70]. This suggests that the composite failed by fiber brittle failure with fiber pull-out and matrix failure. Comparable results for morphology phenomenon were observed for PLA-CNC in some other studies [71].

## 4. Conclusions

The outcome of this research work was the achievement of the uniform dispersion of CNCs in the composites up to 1wt.% as revealed by FESEM, resulting in improvement in their chemical structure, morphologies, thermal and mechanical properties. From TGA/DTG analysis, it was found that CNCs improvised the thermal stability of the composite films and also restricted the crystallization of PLA-PBS blends as demonstrated by XRD and the DSC studies. The FTIR results revealed distinct interaction between the polymers in the blend and no chemical reaction occurred between the constituents of the composites. The tensile strength of the PLA-PBS-CNC composite films were improved upon addition of CNCs because of their homogeneous dispersion as observed from FESEM, leading to effective load transfer and distribution. Further, the tensile modulus increased upon addition of CNCs, making the composite films stiffer; however, the elongation at break decreased insignificantly due to reduction in flexibility of composites upon addition of CNCs. The highest values of tensile strength and tensile modulus approximately 93 MPa and 7600 MPa were obtained for composite with 1wt.% of CNC. Thus, the improved properties of polymer matrix upon addition of CNCs (extracted from bamboo fiber) demonstrate their advantageous features in development of higher strength sustainable polymers as replacement to conventional plastics and material for packaging applications. The novelty of this work was the inclusion of high quality CNCs extracted from bamboo fiber in PLA-PBS polymer blend with enhanced characteristics and properties. Further studies involving combination of techniques or some novel techniques are required for achieving better dispersion of CNCs in order to improve the thermal stability as well as the strength of such bio-composites to broaden their scope in various applications.

## Figures and Tables

**Table 1:** Composition of PLA-PBS-CNC Composites.

S.No	Samples	PLA (wt.%)	PBS (wt.%)	CNC
1.	N1	80	20	0
2.	N2	79.5	20	0.5
3.	N3	79.25	20	0.75
4.	N4	79	20	1
5.	N5	78.5	20	1.5

**Table 2:** TGA and DTG Results of PLA/PBS/CNC Composites.

Samples	T_i_^a^ (°C)	T_50%_^b^ (°C)	T_max_^c^ (°C)	T_f_^d^ (°C)	W_i_^e^ (°C)	W_max_^f^ (%)	W_residue_^g^ (%)
N1	331.7	359.42	366.08	387.3	98.7	22.15	0.562
N2	331.2	363.87	364.37	393.6	98.8	23.91	1.172
N3	334.1	362.94	366.64	391.4	99.3	22.4	0.368
N4	332.1	361.52	363.24	390.8	98.9	23.44	1.068
N5	330.4	361.48	367.52	390.1	98.7	23.68	1.236

^a^ TGA, initial degradation temperature; ^b^ TGA, 50% degradation temperature; ^c^ DTG, peak temperature; ^d^ TGA, final degradation temperature; ^e^ TGA, maximum weight loss; ^f^ DTG, maximum weight loss; ^g^ TGA, char residue weight.

**Table 3:** DSC Results of PLA/PBS/CNC Composites.

Sample	Tg (°C)	Tm (°C)	ΔH (J/g)	Tm1 (°C)	ΔH (J/g)	Tm2 (°C)	ΔH (J/g)
N1	58.49	114.1	7.28	147.2	6.94	153.59	1.04
N2	61.43	113.91	8.06	146.17	7.57	152.87	3.66
N3	63.15	114.5	8.4	147.08	8.33	153.60	4.64
N4	61.73	114.02	10.13	146.43	7.66	153.15	3.02
N5	62.89	113.25	13.52	146.85	7.35	152.56	3.83

**Table 4:** Experimental Results for Tensile Strength, Tensile Modulus and Elongation at Break of Composites.

Samples	Tensile Strength (MPa)	Tensile Modulus (MPa)	Elongation at Break (%)
N1	75.6 ± 0.747	3200 ± 73.59	17.5 ± 0.1256
N2	74.6 ± 0.554	3975 ± 131.49	16.35 ± 0.1039
N3	85.1 ± 0.569	6925 ± 124.39	15.25 ± 0.1041
N4	92.6 ± 0.607	755 ± 144.34	12.9 ± 0.3446
N5	64.6 ± 0.480	3275 ± 85.39	12.45 ± 0.1553

## Data Availability

The data presented in this study are available on request from the corresponding author.
